# Digital twins for lifespan prediction of used storage racks

**DOI:** 10.1038/s41598-026-56844-4

**Published:** 2026-06-16

**Authors:** Zhuming Bi, Donald Mueller, Bin Chen, Chaomin Luo, Muzi Li

**Affiliations:** 1 Department of Civil and Mechanical Engineering, Purdue University Fort Wayne, Fort Wayne, IN 46805 USA; 2 Department of Electrical and Computer Engineering, Purdue University Fort Wayne, Fort Wayne, IN 46805 USA; 3 Electrical and Computer Engineering, Mississippi State University, Starkville, MS 39762 USA; 4 Google GenAI Infra Team, Seattle, WA USA

**Keywords:** Sustainable manufacturing (SM), Reconfigurable manufacturing systems (RMSs), Digital twins (DT-I), Digital triads (DT-II), Finite element analysis (FEA), Storage racks, Lifespan prediction, Factor of safety, Energy science and technology, Engineering, Materials science

## Abstract

Digital twins (DTs) are vital tools to (1) model products or systems at their design stages and (2) monitor systems’ conditions and predict their lifespans in their deployment stages. However, not many real-world case studies of DTs have been reported in literature to show significant benefits to *Small and Medium sized Enterprises* (SMEs). In this paper, DTs are proposed to predict the lifespans of used resources to pursue the sustainability of *Reconfigurable Manufacturing Systems* (RMSs). To illustrate the potential applications of DTs in enhancing the sustainability of real-world SMEs, a *Technical Assistive Project* (TAP) for the assessment of lifespans of materials storage racks is presented. The background and data collection methods are introduced, and digital models for 4 selective racks are developed to evaluate their *Factor of Safety* (FoS) subject to the worst loading scenarios. Parametric design studies are defined and conducted to investigate the dependence of FoS on varying loads. The results support managers’ scientific decision-makings on reusing and recycling manufacturing resources in Sustainable Manufacturing.

## Introduction

With a growing concern on deteriorated environment, society, and economy, pursuing *Sustainable Manufacturing* (SM) becomes crucial for enhancing economic efficiency, reducing adverse environmental impact, and improving quality of life of people^1–3^. SM requires maximizing the uses of manufacturing resources by redesigning, reusing, and recycling in their lifecycles^4–10^. SM are enabled by the digital transformation with numerous information technologies including *digital twins* (DT-I), *digital triad* (DT-II)^11,12^, *big data analytics* (BDA), *Artificial Intelligence* (AI), *Internet of Things* (IoT)^13,14^, and Blockchain Technologies (BCT)^15–17^. In this paper, the uses of DT-I in SM are focused.

When manufacturing businesses are digitized, DT-Is are crucial to model physical entities with a high-level fidelity (Wagg et al. 2020). DT-Is support the circular economy in the sense that a product or system can be modelled or prototyped virtually to minimize invisible waste in creating engineering innovations^18^. DT-Is support the *First-Time-Right* (FTR) strategies^19^ in transforming a virtual model into its physical model. In deploying certain products or systems, its DT-I supports real-time monitoring to sustain efficient operations, improve productivity, and provide predictive maintenance for an extended lifespan. Since the concept of digital twins was introduced in early 2000, DT-I relevant technologies have evolved greatly over years,many concepts including *digital twinning*, *virtual engines, digital counter parts*, *intelligent prognostics* and digital triads (DT-II) have been proposed to advance digital technologies. More importantly, DT-I relevant technologies have been integrated with other newly developed tools such as *Edge Computing* (EC), *Cloud Computing* (CC), and AI to support data-driven decision-making activities in a sustainable manufacturing system a highly turbulent business environment. However, not many real-world case studies of DTs have been reported in literature to show significant benefits to *Small and Medium sized Enterprises* (SMEs).

Recently, there is a growing trend is to incorporate generative AI (genAI) in engineering innovation^20^. However, genAI tools are mostly data-driven while engineering systems are governed by physical laws^21^. There are the emerging needs to integrate physical laws with data-driven Machine Learning (ML) in modelling and simulation physical systems. Especially, it will be promising to obtain and utilize reliable and accurate data from physical-based methods in genAI tools to meet the strict requirements of trustworthiness and reliability in engineering applications^22^.

In this paper, DTs are proposed to predict the lifespans of used resources to pursue the sustainability of *Reconfigurable Manufacturing Systems* (RMSs). The fully verified raw data obtained from DTs is expected to be utilized by genAI tools to assist SME managers in decision-making supports. To illustrate the potential applications of DTs in enhancing the sustainability of real-world SMEs, a *Technical Assistive Project* (TAP) for the assessment of lifespans of materials storage racks is presented. The rest of the paper is organized as follows. Section "Motivation of proposed study" introduces the background of the research project. Section "Data collected for establishment of digital twins" discusses data collection for the establishment of digital twins. Section "Finite element analysis (FEA) modelling procedure" develops digital twins of materials storage racks, and the models are used to predict their remaining lifespans. Section "Load analyses of racks" creates parametric studies to investigate the dependences of lifespans on varying loads. Section "Summary" summarizes our work with the findings from the developed DT-Is.

## Motivation of proposed study

A *Small and Medium sized Enterprise* (SME) client offers fabrication, machining, welding, and finishing capabilities for various processes and industries globally. The client plans to reuse existing racks from other plants to store steel and aluminum sheets but lacks knowledge to analyze the capabilities and remaining lifespans of their equipment. These racks are constructed from steel with reinforced I-beam (or tube) columns and steel tubes welded to the columns in a cantilever arrangement. In fact, these racks have been used to store metal sheets for many years, while the materials to be stored have gotten larger in the laser few years.

The information for original designs and fabrications of these racks was not available, and there were safety concerns if these old racks were used continuously at the facility. In this investigation, four storage racks with safety concerns are selected, and corresponding digital twins are developed to determine most allowable loads subjected to given safety criteria. Accordingly, three objectives of the proposed study are to

1. Create digital twins for the selected racks with safety concerns,
2. Incorporate *Computer Aided Engineering* (CAE) models in digital twins to calculate allowable loads of racks under existing conditions,
3. Make the recommendation of using existing racks subjected to safety constraints (i.e., in terms of *Factor of Safety* (FoS)

## Data collected for establishment of digital twins

**Figure 1:**
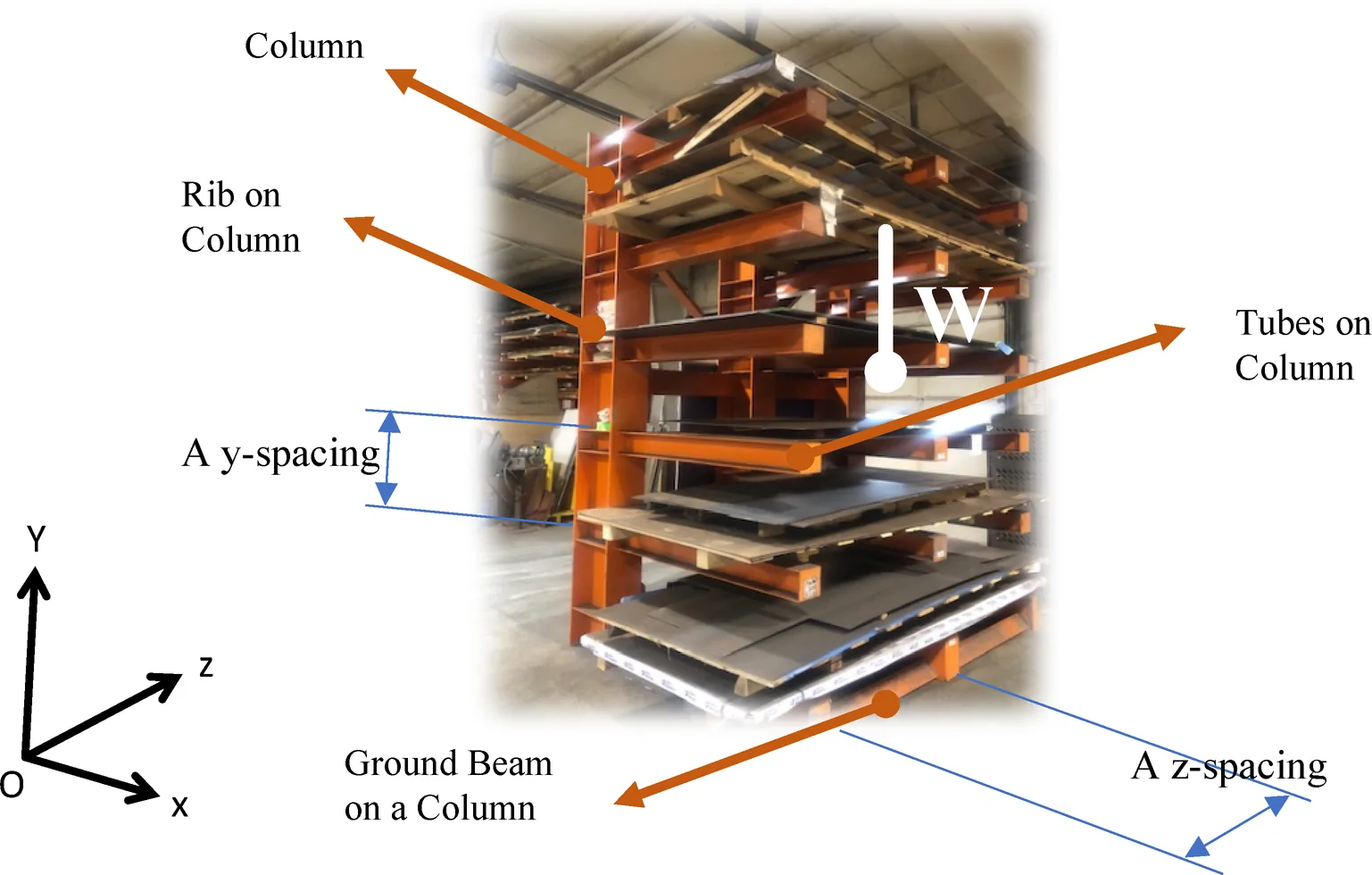
Required dimensions of a rack.

Models and estimation of loads were based upon the information of dimensions, materials, welds, bracing, and configurations that were measured and provided by the client. The structure of rack was measured to obtain as many dimensions as possible. Ideally, all dimensions of structural members in a rack should be measured. These included the main dimensions as below that were illustrated in Fig. 1.

1. **Columns**: (1) number, x-spacing, materials and grades, thickness, height, the type and dimensions over cross-sections, (2) the number of **ribs**, thickness, locations, and **welds** to frames.
2. **Ground beam**/tube on a column: (1) number and z-spacing of contact points, materials and grades, length, the type and dimensions over cross-sections, (2) the configurations of **welds** to frames.
3. **Tubes** on a column: (1) number, the height to the first one, y-spacing of sequential ones, materials and grades, length, the type and dimensions over cross-sections, (2) the configuration of **welds** to frames.

**Figure 2:**
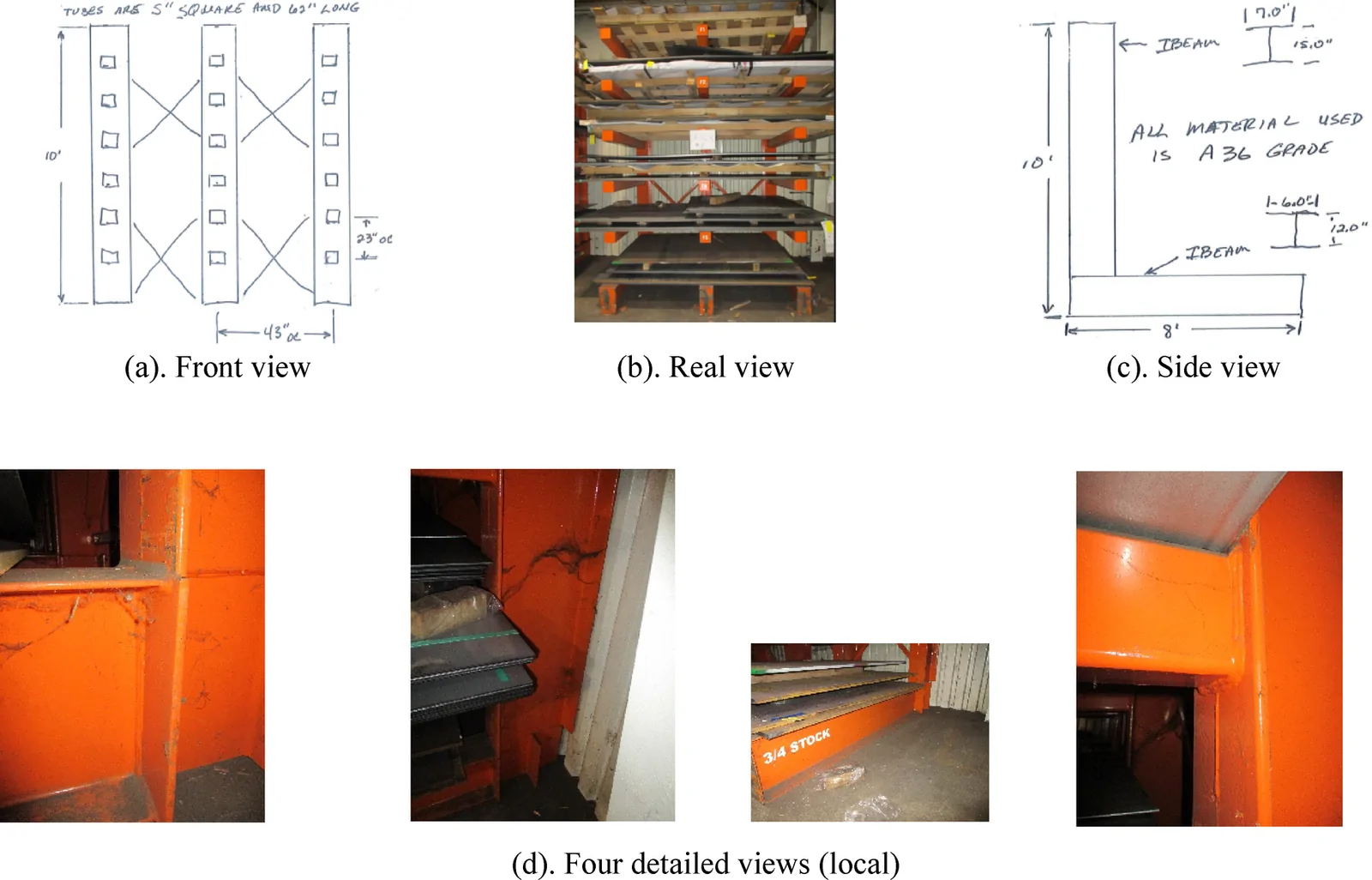
Dimensions of Rack #1.

All racks at the client company were made of A36 steel, Fig. 2 shows the dimensions of Rack #1.

## Finite element analysis (FEA) modelling procedure

In this section, Rack #1 was used as an example to illustrate how a *Computer Aided Engineering* (CAE) model can be developed and used to evaluate the distribution of FoS subjected to given *boundary conditions* (BCs) including geometric constraints and loads. Since the information of weld configurations was unavailable, we assumed that the structures were welded with sufficient strength and the weakest points in a rack with safety concerns were in structural members that were used to build the rack. The procedure of FEA modelling included the following steps.

### Creating a CAD model based on given dimensions

**Figure 3:**
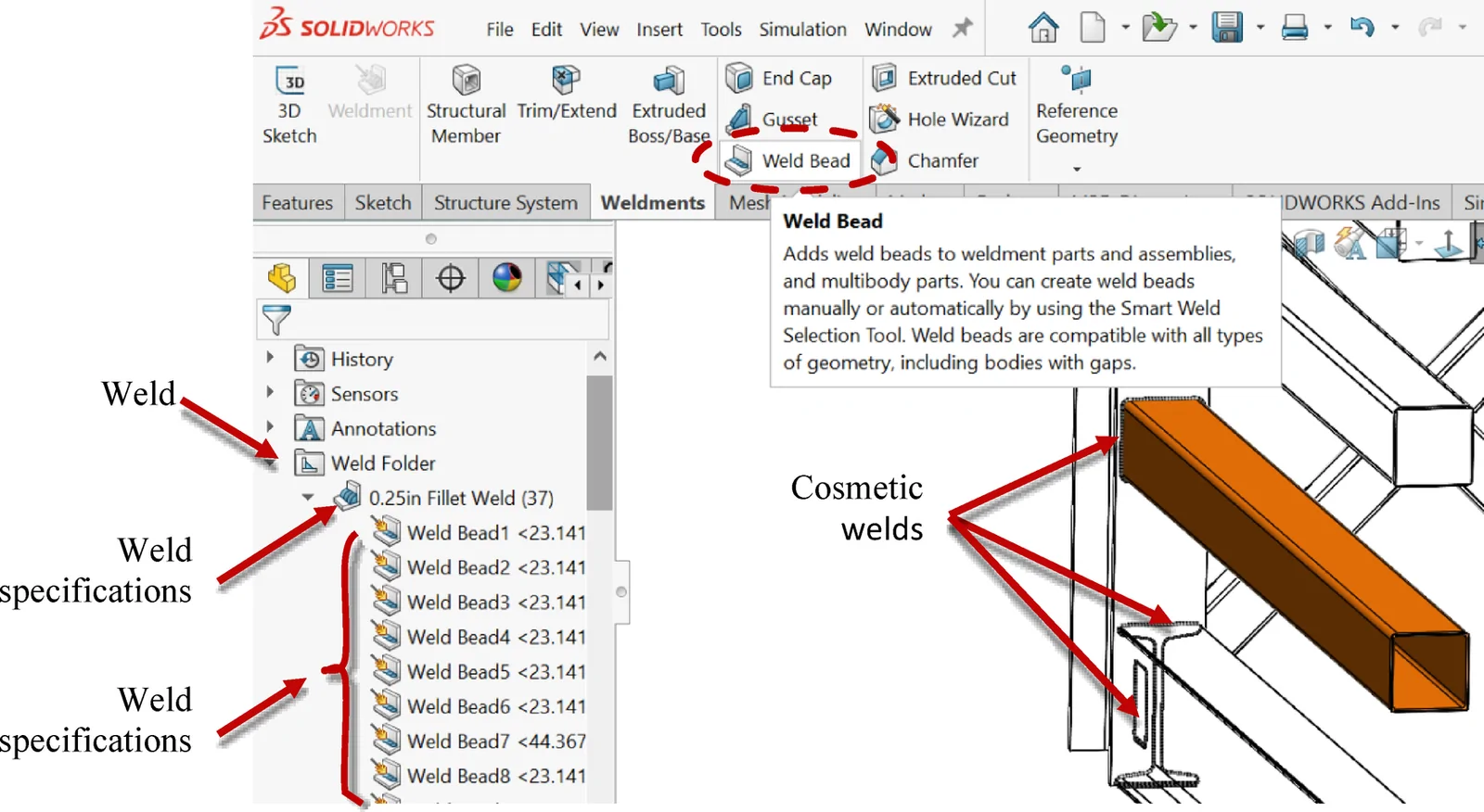
Weld members into a single structure.

The first step was to reverse engineering existing racks. The weldment in SolidWorks was an ideal tool to create a welded structure effectively^23^. Creating a welded structure involves the following step:

1. One or more 2D or 3D sketches were defined to locate structural members.
2. The ‘Structural Member’ tool under the ‘Weldment’ Toolbox is used to create all structure. A section is specified for one or a group of structural members using the design libraries for standardized sections of structure members^24^ When a structural member is not standardized, a closet standard section can be selected, and it can be further edited to customize the depth and width of the profile.
3. Repeat step (2) until all structural members are defined. Then, the ‘Sketches’ and ‘Features’ tools are used to create other customized parts (including caps of structural members), respectively; these parts are included to assemble all members into one structure as shown.
4. Finally, the ‘Weld Bead’ tool under the ‘Weldment’ Toolbox is used to define welds where two structural members are joined. All defined weld beads will be listed in ‘Weld Folder’ of the model tree, and they can be visualized as shown in Fig. 3 by turning on ‘welds’ in ‘Detailing’ of ‘Document Properties’ of ‘Options’.

### Defining an FEA model

**Figure 4:**
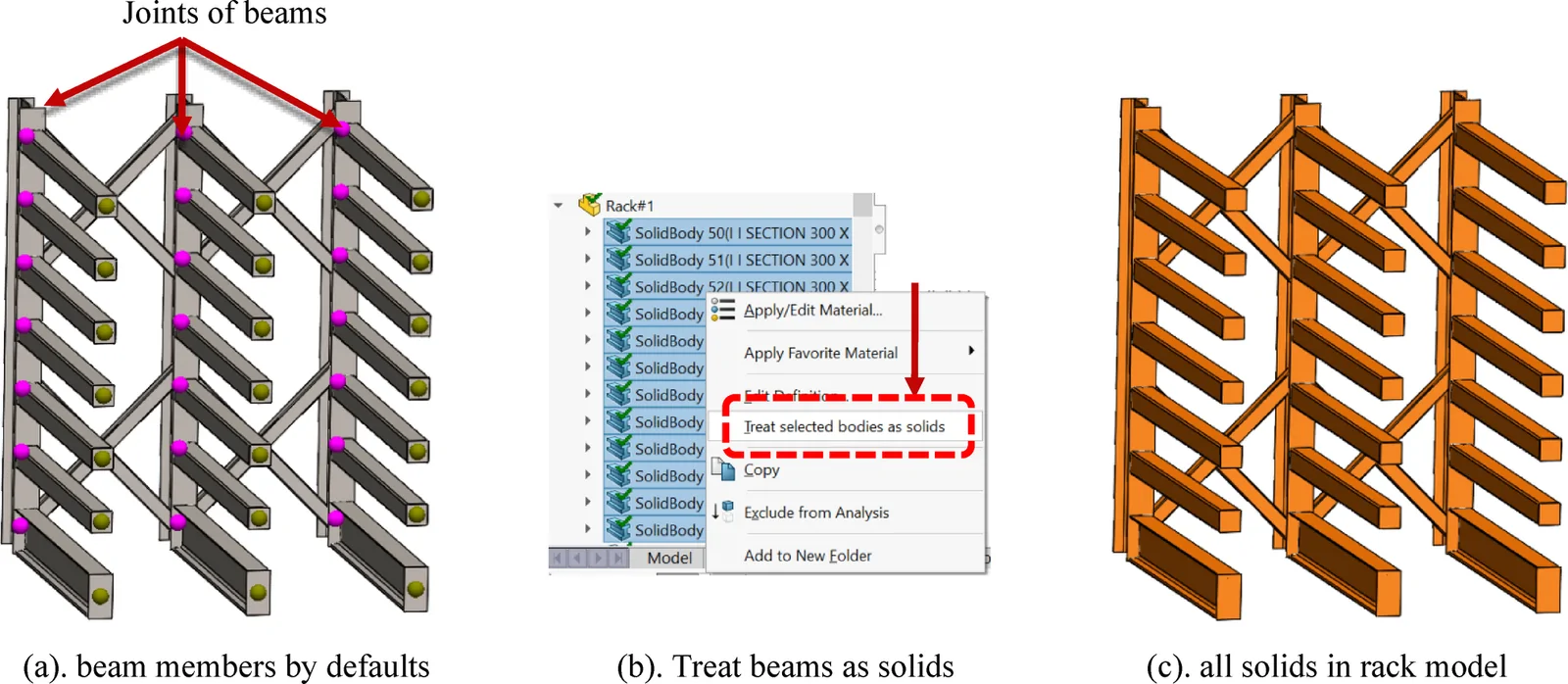
Set all beam members as ‘solids’ in FEA model.

As shown in Fig. 4, an FEA model can be defined directly for a solid model of rack. Since a rack model is created by using a weldment tool, structural members are modelled as ‘beam’ elements by defaults. It will be better to set all ‘beam’ members as ‘solids’ for the purpose of better accuracy in evaluating stress distributions at interfaces.

### Specifying materials properties

All structural members use the same type of materials, i.e., A36 steel. This material is included in the Materials Libraries of SolidWorks. Therefore, the materials can be assigned to all structural members simply by applying ‘ASTM A36’ on all selected solids. The yield strength ($S_{y} = 36.259 (kpsi$)) and tensile strength ($S_{ut} = 58.016 (kpsi$)) are used to evaluate FoS of the rack subjected to different loading conditions.

### Applying ‘mech control’ and creating mesh

**Figure 5:**
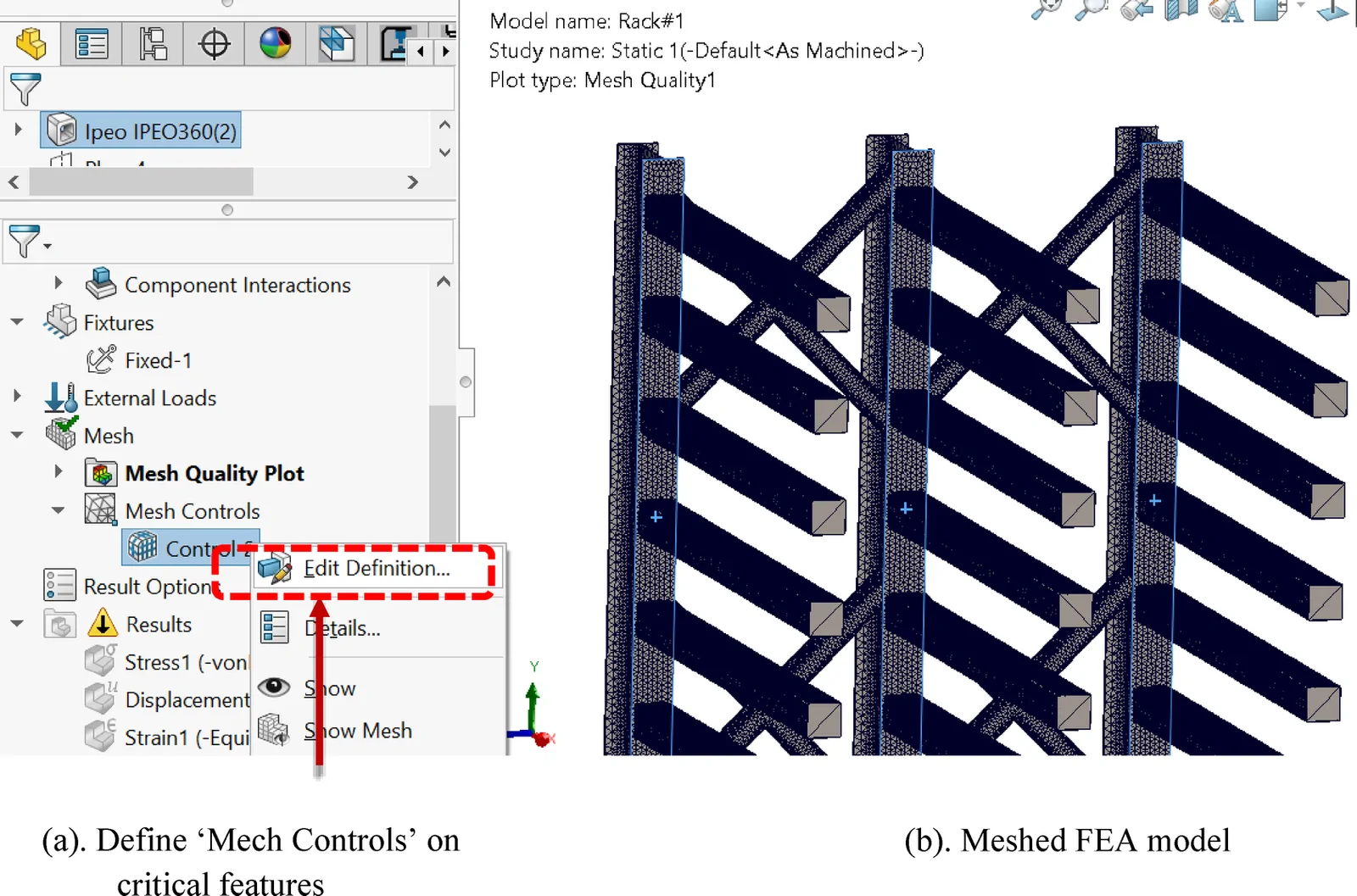
Defining ‘Mesh Controls’ and creating a mesh for solids.

An FEA model discretizes a continuous domain into a discrete domain with the finite numbers of elements and nodes. The accuracy of an FEA solution depends on the sizes and distributions of elements. Generally, the finer elements are, the better accuracy can be achieved at a higher computation cost. For fast computation, element sizes can be set as default that are determined by computer algorithms automatically. When an engineer is interested in stress distributions at specific features of interest, Mesh Controls can be defined by customizing the types and sizes of elements that are used to discretize these features as shown in Fig. 5a. Figure 5b gives an example of successfully meshed FEA model with solids.

### Applying boundary conditions on FEA model

**Figure 6:**
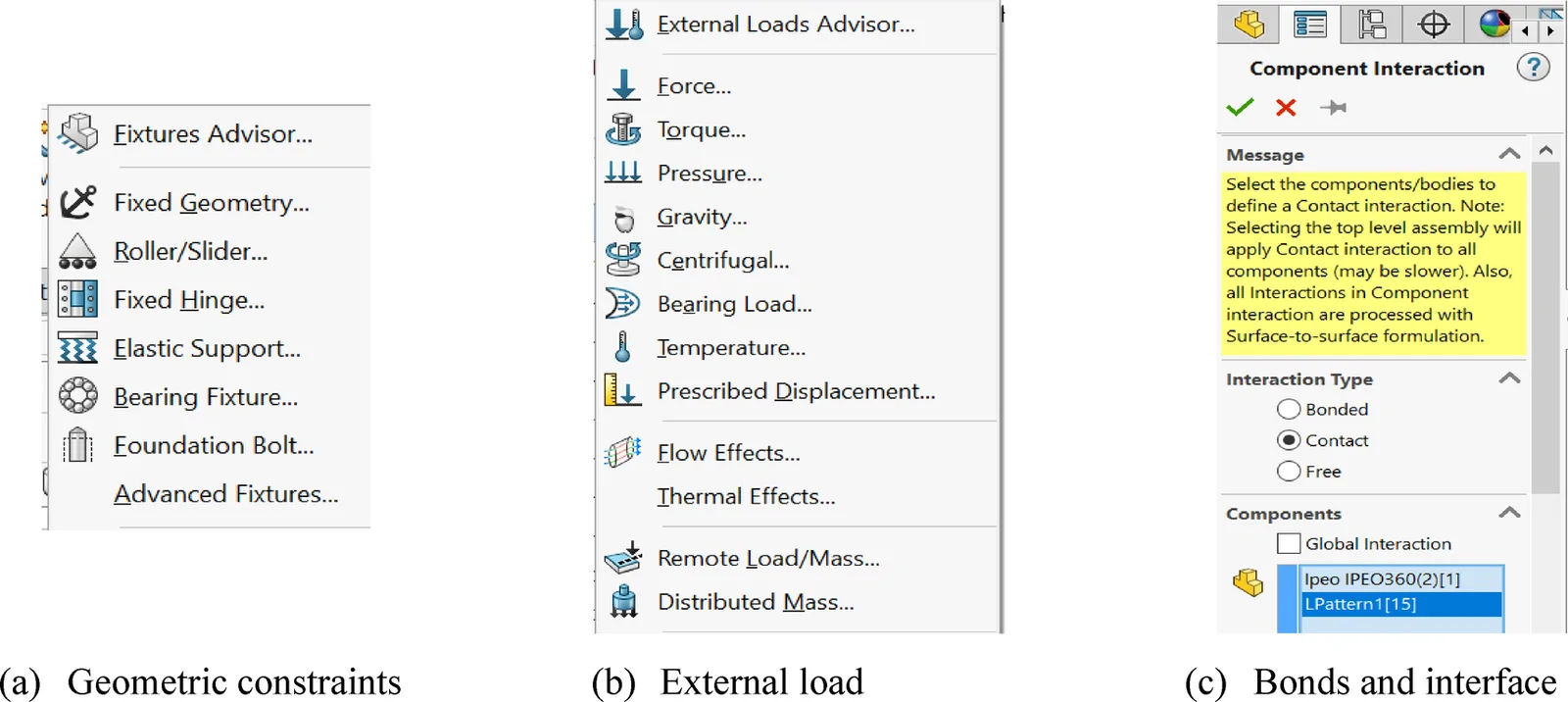
FEA model of Rack #1.

An FEA model must be fully restrained to be solved appropriately. The constraints on an FEA model can be classified into (1) geometric constraints such as fixed geometries and rollers shown in Fig. 6a, (2) the external loads such as gravity, forces, pressures, and remote loads shown in Fig. 6b, and (3) the conditions at interfaces such as ‘bonds’ in Fig. 6c.

**Figure 7:**
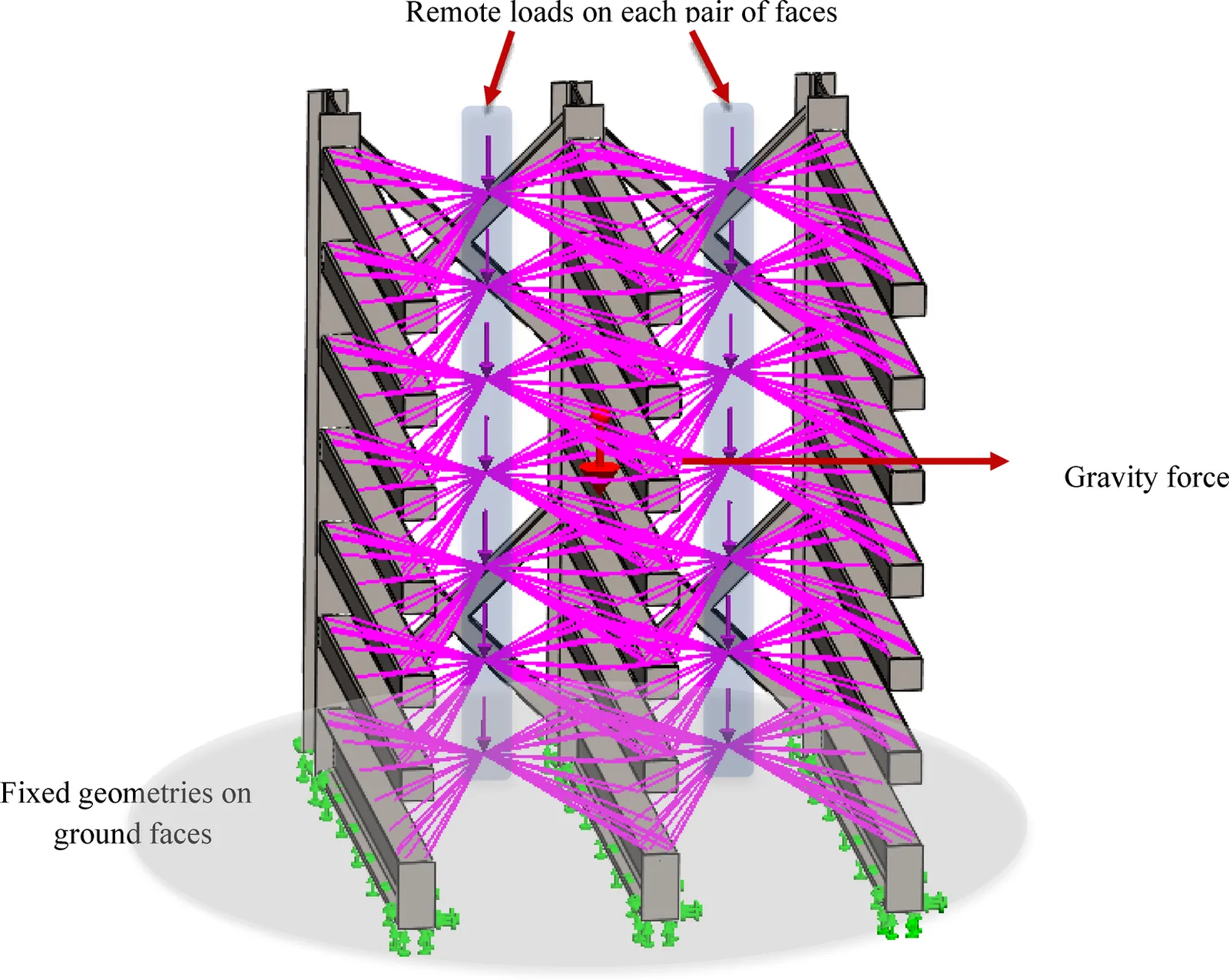
Defining BCs in an FEA model.

Since racks have been used for many years, it would be reasonably assumed that all the welds are produced appropriately with sufficient bonding strengths. Therefore, default ‘global bonds’ are applied as a rack model for item. For geometric constraints in (1), the ‘fixed geometry’ is applied to all surfaces that contact to ground. For external loads in (2), the worst scenario corresponds to the case where all structural members are used to carry the same amount of force in each pair of neighboring structural members at the same height; accordingly, a remote load is defined for each member in a pair of neighboring structural members. Figure 7 shows the FEA with defined BCs.

### Solving FEA model for distributions of stresses, deflections, and FoS

**Figure 8:**
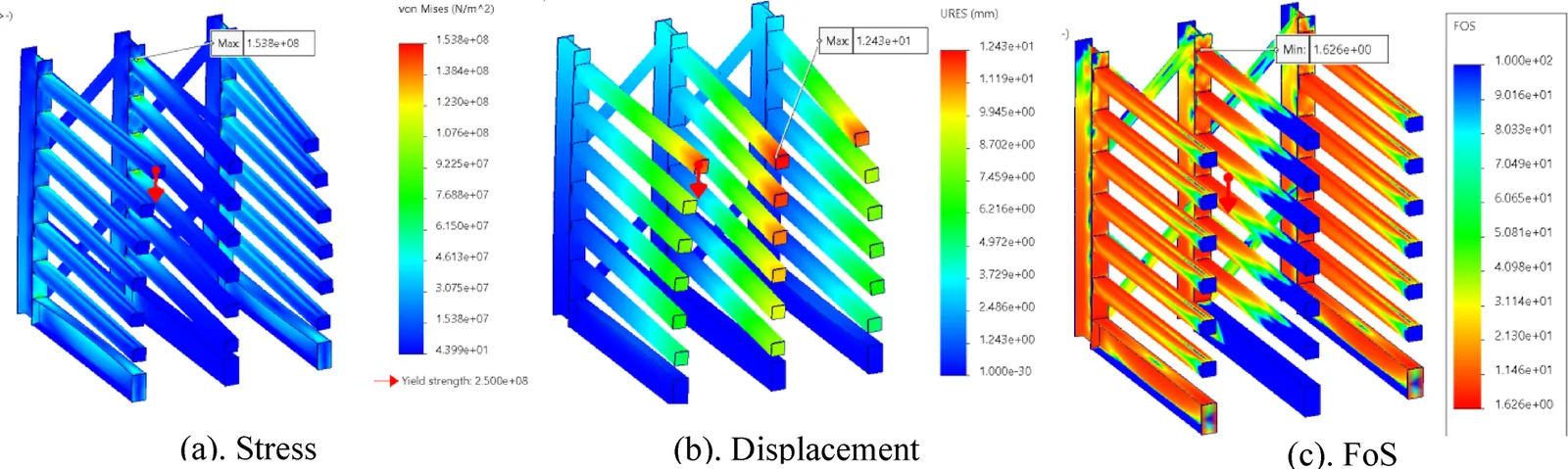
Examples of post-processed outcomes.

The FEA model can be readily solved by clicking ‘Running This Study’ in Simulation. The computation time depends on the size (i.e., the numbers and types of elements and nodes) of the FEA models. Once the solution is obtained, it can be post-processed to retrieve and visualize outputs such as the distributions of stress, deflections, and FoS as shown in Fig. 8a–c, respectively.

### Defining ‘design study’ to investigate impact of loads on FoS

**Figure 9:**
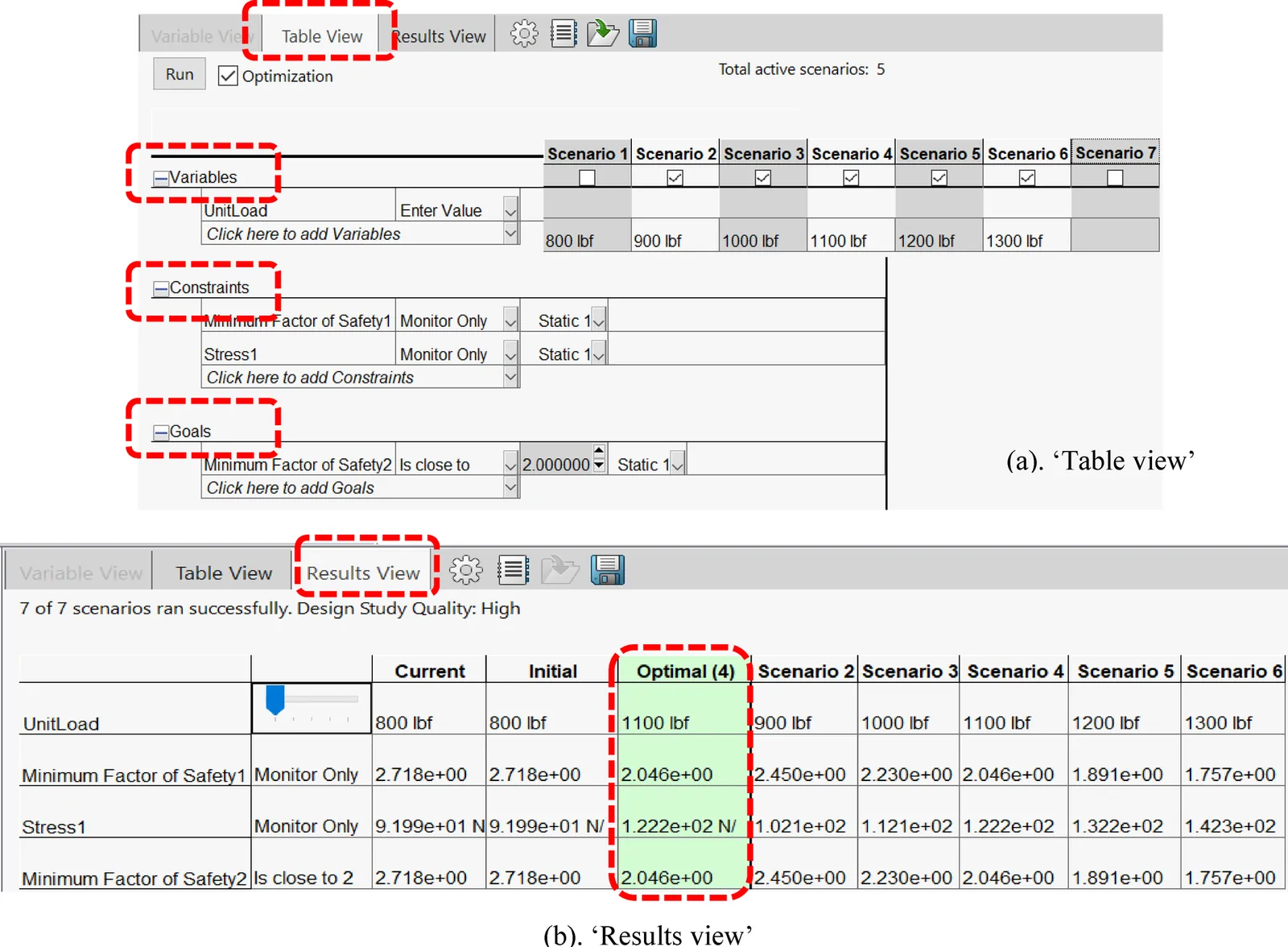
Defining a ‘Design Study’ to assess impact of external load on FoS.

To investigate the impact of the external load on the safety use of a rack, a ‘Design Study’ can be created on an original ‘static’ FEA model. A Design Study defines ‘variables’, ‘constraints’, and ‘goals’ as shown in Fig. 9a, respectively. Any parameters in the model including those for external loads can be changed; the parameters of interest are specified in ‘variables’ with a set of discrete values or with a changing range with given step. In this study, only the amplitude of a unit load is defined as a changeable parameter.

We investigate the impact of the unit load on stresses and FoS of rack. Therefore, the maximum stress and the minimum FoS are defined as measures to be monitored in ‘constraints. Finally, the client is especially interested in the maximum allowable load subjected to specified FoS; therefore, targeted FoS (it is set as ‘2’ in this project) is selected as the output measure in optimization as optimized criterion of ‘is close to’.

### Verifying simulation results

A convergence of a simulation result of a solid mechanics problem can be simplified by (1) treating the whole structure as a free-body or isolating any part or component from the structure and verifying if all forces applied on object are balanced; (2) simplifying a rack as a structure with beam, verifying the shear and moment diagrams by hand calculation.

#### Force balance on structure or object

**Figure 10:**
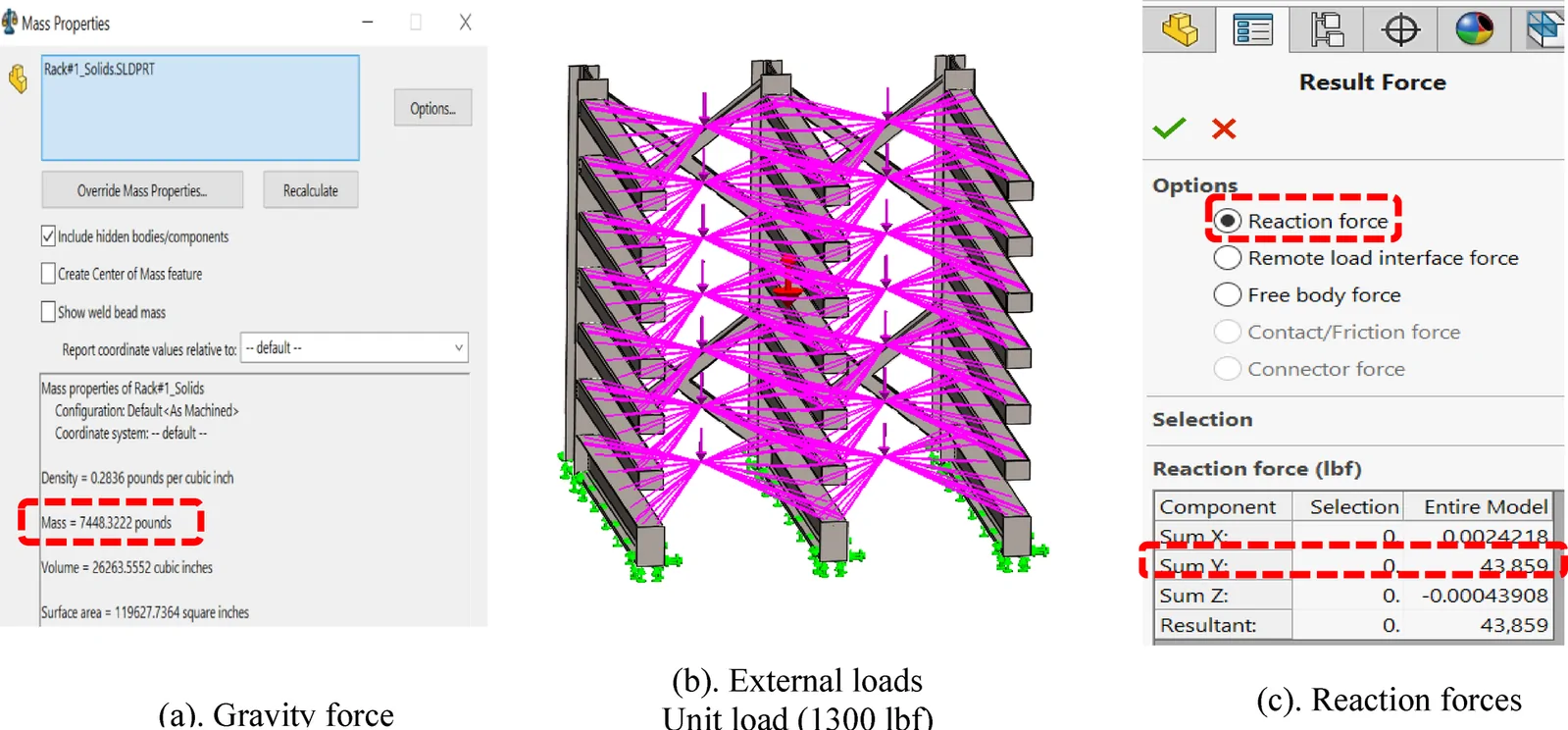
Data collection for verification of simulation results.

Here, the first method on the force balance on the whole structure is used. The structure has the gravity force which can be found by evaluating the mass properties of the whole rack as shown in Fig. 10a.

For the verification of the simulation shown in Fig. 10, one has the residual force of

$$\left\{ {\begin{array}{*{20}l} {R_{x} = 0.010773 lbf} \hfill \\ {R_{y} = 43859 - 7448.3222 - 1300\left( 7 \right)\left( 2 \right)\left( 2 \right) = 10.68 lbf} \hfill \\ {R_{z} = - 0.0019531 lbf} \hfill \\ \end{array} } \right.$$

This verifies the results since the residual forces in all three directions are ignorable in comparison with the amplitudes of external forces.

#### Hand calculation on shear and moment diagrams of object

**Figure 11:**
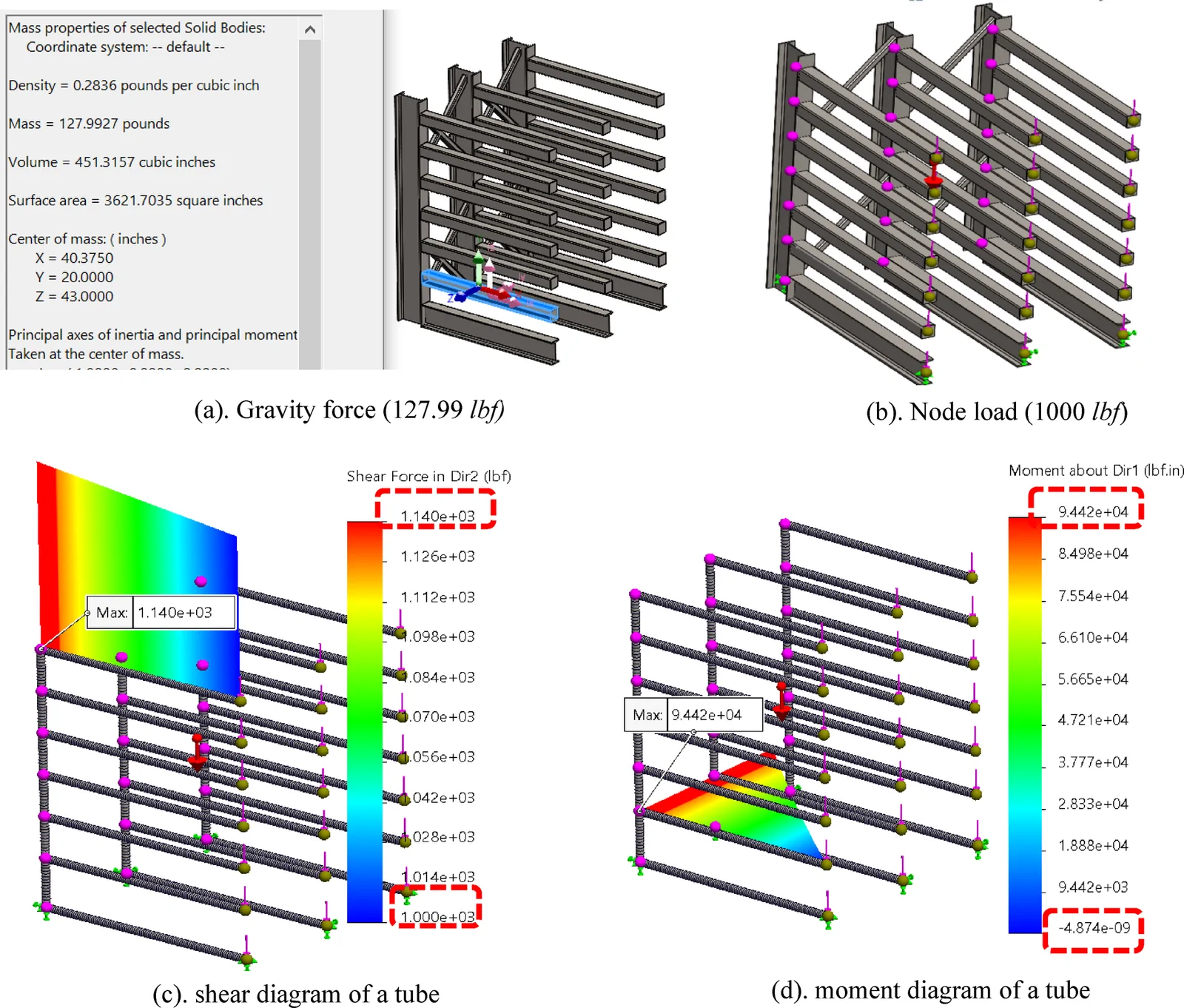
Verifying a model by hand calculation on shear and moment diagrams on object.

Here, we illustrate how a simplified beam structure can be used to verify an FEA model. Since gravity must be taken into consideration, the information of an individual object should be available by evaluation as shown in Fig. 11a. The verification considered applying to a node force 1000 lbf at free end of a tube as shown in Fig. 11b. The shear diagram of a tube from the simulation was shown in Fig. 11c.

The net shear force at the point of interest became,

$$\sum F_{y} = 1140 - 1000 - 127.99 = 12 \left( {lbf} \right)$$

The net moment at the point of interest became,

$$\sum M_{z} = 94420 - 1000\left( {96 - 7.5} \right) - 127.99\frac{{\left( {96 - 7.5} \right)}}{2} = 252.6 \left( {lbfin} \right)$$

It is reasonable approximation by considering the impact of the weight of vertical column was not taken into consideration.

## Load analyses of racks

**Figure 12:**
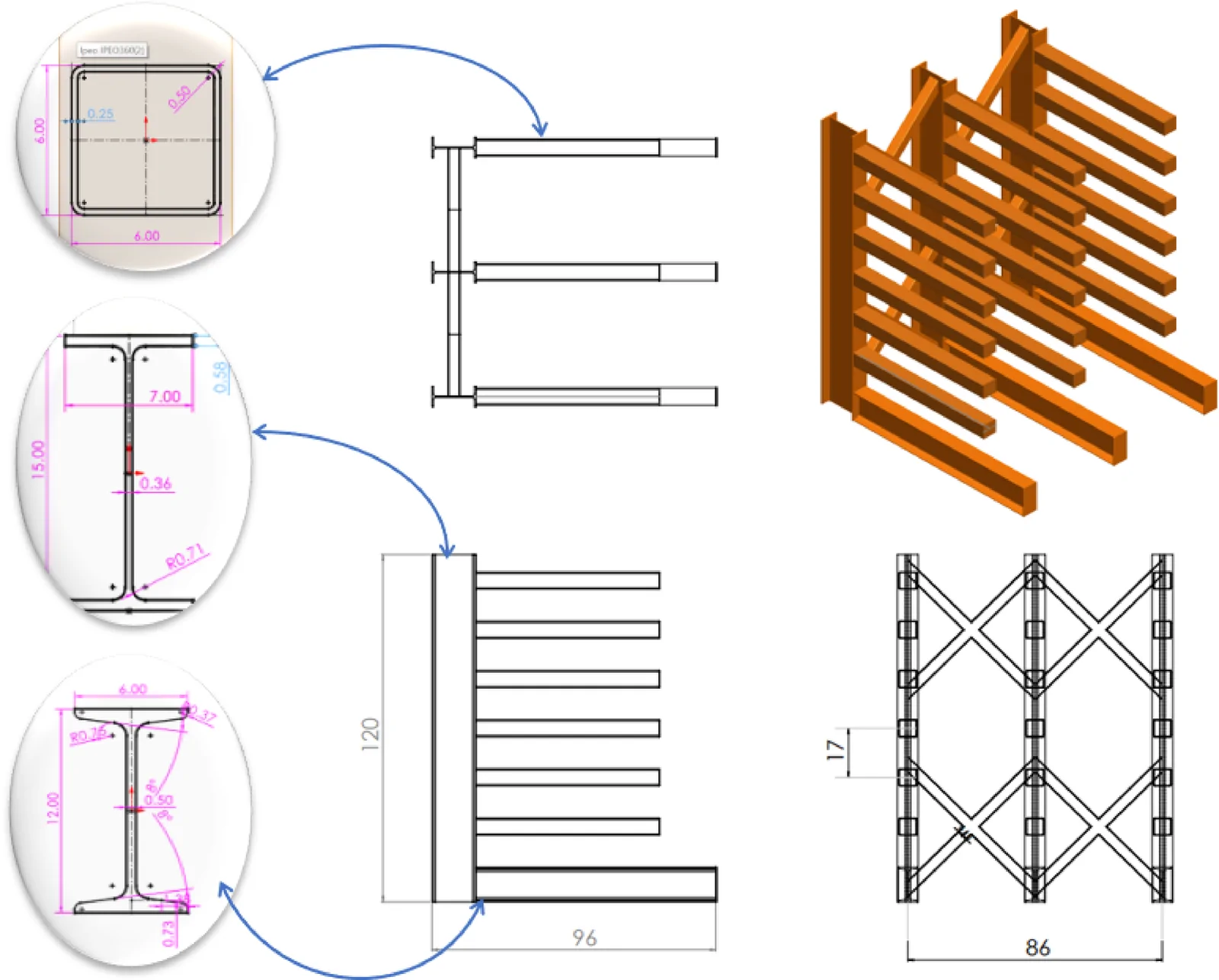
Drawing of rack #1 model.

**Figure 13:**
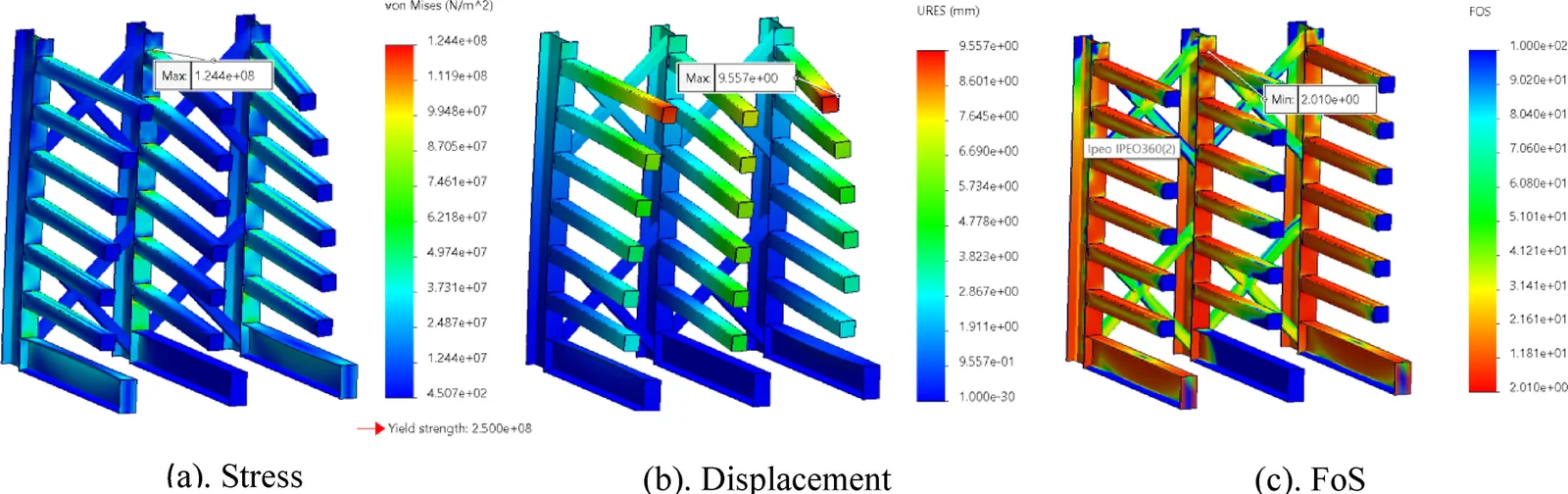
Distributions of stresses, displacements and FoS when unit load = 1200 lbf.

**Figure 14:**
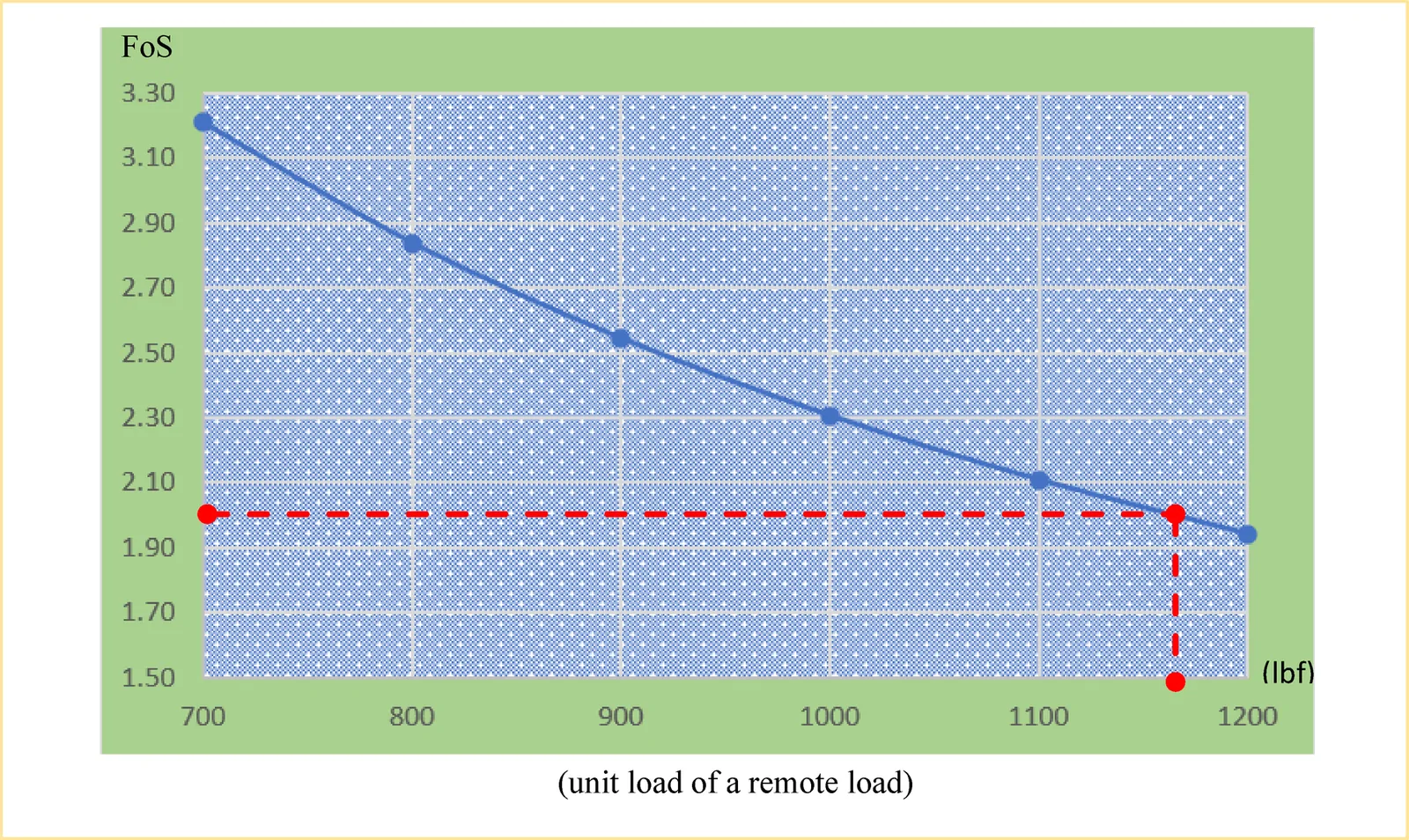
Unit load v.s. FoS for rack #1.

This section takes an example of Rack #1 as example to show the corresponding result simulation. Figure 12 showed the drawing of the CAD model for rack #1 with main dimensions. Figure 13 showed the simulation result subjected to a unit load of 1200 lbf. Table 1 and Fig. 14 showed the correspondence of the unit load and FoS.

**Table 1:** The results of the Design Study (a total of 28 unit loads).

UnitLoad (lbf)	700	800	900	1000	1100	1200
Min FoS	3.21E+00	2.84E+00	2.55E+00	2.31E+00	2.11E+00	1.94E+00
Max Stress (MPa)	7.79E+01	8.81E+01	9.82E+01	1.08E+02	1.19E+02	1.29E+02

## Summary

DT-Is are crucial to modeling physical entities with high-level fidelity. DT-Is support the circular economy in the sense that a product or system can be modelled or prototyped to minimize invisible waste in creating engineering innovations. DT-Is support the FTR strategies in transforming a virtual model into its physical model. In deploying certain products or systems, its DT-I supports real-time monitoring to sustain efficient operations, improve productivity, and provide predictive maintenance for an extended lifespan. However, not many real-world case studies of DTs have been reported in literature to show significant benefits to SMEs. In this paper, DTs are proposed to predict the lifespans of used resources to pursue the sustainability of RMSs in a case study.

This presented work was the outcome of an industry project with an SME client. The information of four racks was collected by the client as raw data for the authors to digitize selected materials storage racks. Digital twins were created for these products, and FEA simulation models were incorporated to analyze FoS of these products subjected to the loads in the worst scenarios. Moreover, design studies were conducted to correspond external loads to FoS of racks. The results were used as the guide to recommend the maximum allowable loads subjected to given safety criteria in the form of FoS. The proposed method is expected to be generalized and used by other SME clients to reuse existing manufacturing resources in pursuing systems’ sustainability.

While the case study is specifically developed to predict the lifespans of used storage racks for sustainable manufacturing, the proposed methodology is generic and extendable to other applications when ante-hoc interpretability and trustworthy of the outcomes from genAI become essential to fully utilize these AI-generated outcomes. By using FEA tools and DTs to generate raw data that obeys physical laws, SME managers can make transparent and justifiable decisions without the needs of deep understanding of how ML reasoning processes are performed to reach these decisions by AI-driven tools. Our future plan on this research topic is to incorporate DTs in a genAI model to assist SME managers in redesigning and reusing manufacturing resources for sustainable manufacturing. By all means, using the developed DTs allows us to generate trustworthy, sufficient, and reliable data since the data is generated from fully verifiable physical models. Using reliable data in genAI can address some significant challenges genAI in engineering applications such as hallucination, inconsistency, and lacks of interpretability, transparency, verification and validation^25^. On the other hand, incorporating genAI will further empower the proposed DTs to automate follow-up decision makings and improve efficiency of DT-driven product life-cycle management (PLM)^26^.

## Data Availability

The experimental data and the simulation results that support the findings of this study are available upon request from the corresponding author.

## References

[1] Karuppiah, K., Sankaranarayanan, B. & Lo, H. W. A systematic literature review on the evolution of sustainable manufacturing practices: Key findings and implications. *Clean. Eng. Technol.* **22**, 100798 (2024).

[2] Khan, A. A. & Hasan, M. Smart hybrid manufacturing: A combination of additive subtractive, and lean techniques for agile production systems. *J. Sustain. Dev. Policy* **2**(4), 174–217 (2023).

[3] Leng, J. et al. Review of manufacturing system design in the interplay of industry 4.0 and industry 5.0 (Part I): Design thinking and modeling methods. *J. Manuf. Syst.* **76**, 158–187 (2024).

[4] Afify, M., Moubachir, Y., Guennoun, Z. & Hassar, J. A hybrid CAD/CAE/CAM project based laboratory framework integrated topology optimization and laser powder bed fusion for engineering education. *Comput. Appl. Eng. Educ.* **23**(3), e22745 (2024).

[5] Bilal, H., Bi, Z., Younis, N. & Abu-Mulaweh, H. Human machine interaction through brain computer interface. In *2025 IEEE International Conference on Robotics and Biomimetics (ROBIO)*, Chengdu, China, pp. 2476–2481 (2025).

[6] Geissdoerfer, M., Pierani, M. P. P. & Pigosso, D. C. A. Circular business models: a review. *J. Clean Prod.* **277**, 123741 (2020).

[7] Pang, S., Guo, F., Zhang, B., Huang, H., Pan, X. & Shang, W. High-stiffness path planning for 7-DOF cable-driven manipulators in single and dual-arm configurations. In *2025 IEEE/RSJ International Conference on Intelligent Robots and Systems (IROS)*, Hangzhou, China, 2025, pp. 17785–17790 (2025)

[8] Wu, H. et al. Elastic contact analysis and fatigue life prediction of ball screws in automotive REPS systems. *Int. J. Mech. Based Design Struct. Mach.* **54**(1), 2559419 (2026).

[9] Xu, L., Li, G. & Bi, Z. M. Digital twins to predict crack propagation of engineering materials under different loads for sustainable products. *Machines* **12**(2), 125. 10.3390/machines12020125 (2024).

[10] Zhang, K., Zhang, Y. & Gao, H. Transfer learning-enabled multiobjective optimization for adjustable aircraft assembly scheduling. *IEEE Trans. Ind. Inf.* **22**(3), 2517–2529 (2025).

[11] Lei, T., Luo, C., Ball, J. E. & Bi, Z. A hybrid fireworks algorithm to navigation and mapping, Handbook of Research on Fireworks Algorithms and Swarm. *Intelligence*10.4018/978-1-7998-1659-1.ch010 (2020).

[12] Luo, H. et al. A short-term energy predicton system based on edge computing for smart city. *Futur. Gener. Comput. Syst.* **101**(2019), 444–457 (2019).

[13] Wang, C., Bi, Z. M. & Xu, L. D. IoT and cloud computing in automation of assembly modeling systems. *IEEE Trans. Ind. Inform.* **10**(2), 1426–1434 (2014).

[14] Xu, L. D., Xu, E. L. & Li, L. Industry 4.0: state of the art and future trends. *Int. J. Prod. Res.* **56**(8), 2941–2962 (2018).

[15] Chavamakui, D. H. T., Xu, L. D., Bi, Z. M., Shankar, A. & Dhiman, G. A systematic literature review on resilient digital transformation, examining how organizations sustain digital capabilities. *HighTech Innov. J.* **6**(2), 687–722 (2025).

[16] Viriyasitavat, W., Xu, L. D., Niyato, D., Bi, Z. M. & Hoonsopon, D. Applications of blockchain in business processes: A comprehensive review. *IEEE Access* **10**, 118900–118925 (2022).

[17] Viriyasitavat, W., Xu, L. D., Dhiman, G. & Bi, Z. M. Blockchain-as-a-service for business process management: Survey and challenges. *IEEE Trans. Serv. Comput.* **16**(3), 2299–2314 (2023).

[18] Ghasemzadeh, K., Jafari, M., Torabi, T., Amire, A. & Iulianelli, A. Hybrid models, digital twins, and digital shadows for sustainable membrane technologies: A Critical review. *Adv. Membr.* **8**, 100219 (2026).

[19] Reisch, R. T., Janisch, L., Tresselt, J., Kamps, T. & Knoll, A. Prescriptive analytics – A smart manufacturing system for first-time-right printing in wire arc additive manufacturing using a digital twin. *Procedia CIRP* **118**(2023), 759–764 (2023).

[20] Carvello, V. Generative AI and the future of innovation management: A human centered perspective and an agenda for future research. *J. Open Innov. Technol. Market Compl.* **11**(1), 100456 (2025).

[21] Mariani, M. & Dwivedi, Y. K. Generative artificial intelligence in innovation management: a preview of future research developments. *J. Bus. Res.* **175**, 114542 (2024).

[22] Yu, L., Alegroth, E., Chatzipetrou, P. & Gorschek, T. Measuring the quality of generative AI systems: Mapping metrics to quality characteristics—Snowballing literature review. *Inf. Softw. Technol.* **186**, 107802 (2025).

[23] SolidWorks (2021). Weldments, https://help.solidworks.com/2021/english/solidworks/sldworks/c_weldments_overview.htm. Accessed on June 4, 2024.

[24] Javelin (2023). Download SolidWorks weldment profiles, https://www.javelin-tech.com/blog/2023/10/download-solidworks-weldment-profiles/. Accessed on June 4, 2024.

[25] Esposito, M. et al. Generative AI for software architecture: Applications, challenges, and future directions. *J. Syst. Softw.* **231**, 112607 (2026).

[26] Chiaro, D., Qi, P., Pescapè, A. & Piccialli, F. Generative AI-empowered digital twin: A comprehensive survey with taxonomy. *IEEE Trans. Ind. Inf.* **21**(6), 4287–4295 (2025).

